# Interplay of TGFβ signaling and microRNA in thyroid cell loss of differentiation and cancer progression

**DOI:** 10.20945/2359-3997000000172

**Published:** 2019-08-28

**Authors:** Cesar Seigi Fuziwara, Kelly Cristina Saito, Edna Teruko Kimura

**Affiliations:** 1 Universidade de São Paulo Departamento de Biologia Celular e do Desenvolvimento Instituto de Ciências Biomédicas Universidade de São Paulo São Paulo SP Brasil Departamento de Biologia Celular e do Desenvolvimento, Instituto de Ciências Biomédicas, Universidade de São Paulo, São Paulo, SP, Brasil

**Keywords:** Thyroid cancer, microRNA, TGFβ, EMT, thyroid cell differentiation

## Abstract

Thyroid cancer has been rapidly increasing in prevalence among humans in last 2 decades and is the most prevalent endocrine malignancy. Overall, thyroid-cancer patients have good rates of long-term survival, but a small percentage present poor outcome. Thyroid cancer aggressiveness is essentially related with thyroid follicular cell loss of differentiation and metastasis. The discovery of oncogenes that drive thyroid cancer (such as *RET*, *RAS*, and *BRAF*), and are aligned in the MAPK/ERK pathway has led to a new perspective of thyroid oncogenesis. The uncovering of additional oncogene-modulated signaling pathways revealed an intricate and active signaling cross-talk. Among these, microRNAs, which are a class of small, noncoding RNAs, expanded this cross-talk by modulating several components of the oncogenic network – thus establishing a new layer of regulation. In this context, TGFβ signaling plays an important role in cancer as a dual factor: it can exert an antimitogenic effect in normal thyroid follicular cells, and promote epithelial-to-mesenchymal transition, cell migration, and invasion in cancer cells. In this review, we explore how microRNAs influence the loss of thyroid differentiation and the increase in aggressiveness of thyroid cancers by regulating the dual function of TGFβ. This review provides directions for future research to encourage the development of new strategies and molecular approaches that can improve the treatment of aggressive thyroid cancer.

## THYROID CANCER

Thyroid cancer is the most common malignancy of the endocrine system, and its global incidence has increased in recent years; 52 070 new cases are expected to occur in the United States in 2019 (1,2). The majority of thyroid cancers originate from the follicular cells; in terms of histological and clinical behavior, these cancers are classified as well-differentiated, poorly differentiated and undifferentiated (3). Among differentiated thyroid cancers, papillary thyroid cancer (PTC) comprises more than 80% of cases; the remainder consists of follicular thyroid cancer. Although less frequent (2%-5% of cases), the undifferentiated thyroid cancer or anaplastic thyroid cancer (ATC) is the most aggressive and lethal type of thyroid cancer (3,4).

The main oncogenic alterations of thyroid cancer occur in genes that are aligned with the MAPK pathway. *BRAF* and *RAS* mutations, and *RET/PTC* rearrangements can impair the differentiation of the thyroid follicular cells and lead to PTC oncogenesis due to the constitutive activation of MAPK/ERK signaling (4,5). The acquisition of additional molecular alterations in coding genes (e.g., *PIK3CA* and *AKT1*) may also contribute to loss of differentiation, refractoriness to radioiodine therapy, and aggressive behavior (6). Additionally, the oncogenic activation of the MAPK pathway triggers the deregulation of microRNAs (miRNAs), which comprise a class of small noncoding RNAs that exert a potent inhibitory effect on protein expression at the posttranscriptional level. Because miRNAs modulate targets in several oncogenic pathways (7), they expand the network of oncogene-modulated genes in thyroid cells’ functioning and biology.

## MICRORNA AND THYROID FOLLICULAR CELL DIFFERENTIATION

Thyroid-gland epithelial cells (known as thyrocytes or thyroid follicular cells) are organized in follicles; the previously synthesized thyroid hormones are confined to the follicular lumen. These cells have the unique ability to incorporate the iodine molecule as a thyroid hormone compound (8). The thyrocytes’ differentiated status (i.e., the ability to trap iodine and produce thyroid hormones) is associated with the expression of genes related to iodine metabolism such as *NIS* (sodium iodide symporter), *TPO* (thyroperoxidase), *TG* (thyroglobulin), and *DUOX* (dual oxidase); all of these are directly involved in trapping iodine and metabolizing it into the thyroid hormones. These genes are under the regulation of thyroid transcription factors (TFs) such as NKX2-1 (NK2 homeobox 1, previously known as TTF1), FOXE1 (forkhead box E1, also known as TTF2), and PAX8 (paired box 8), which are uniquely co-expressed in the thyroid gland (9).

Moreover, thyrocytes’ function is regulated by the pituitary thyroid-stimulating hormone via its receptor (*TSHR*), which is expressed in the basolateral membrane of thyroid follicular cells. TSH signaling activation induces thyroid cell differentiation by directly stimulating the expression of genes such as *NIS*, *TPO*, and *TG* through the binding of cAMP to its responsive element in the promoter region of these genes (10).

Since the discovery of miRNA molecules in 1993, more than 2000 human miRNA genes have been described in human genome (miRBase, release 22.1) (11). Mature miRNAs consist of small, noncoding RNAs with ~22nt that posttranscriptionally control gene expression by binding to the 3’ untranslated region within the target mRNAs (12,13). The overall effect of miRNA binding is a reduction in protein levels due to impairment of the ribosome assembly or the induction of mRNA decay (13). Overexpression of miRNA represses the expression of target mRNA, and conversely, the loss of miRNA expression enhances the expression of mRNA. Thus, unbalanced miRNA expression is involved in the pathogenesis of human diseases, including cancer, for which unbalanced miRNA acts in an oncogenic or tumor-suppressive fashion. In terms of bioinformatics, miRNAs regulate more than 60% of protein-coding genes’ mRNAs (based on miRNA seed-sequence conservation) (12); this indicates that these noncoding RNAs could regulate all cellular processes, including thyroid-cell differentiation and tumorigenesis (7,15).

**Figure 1:**
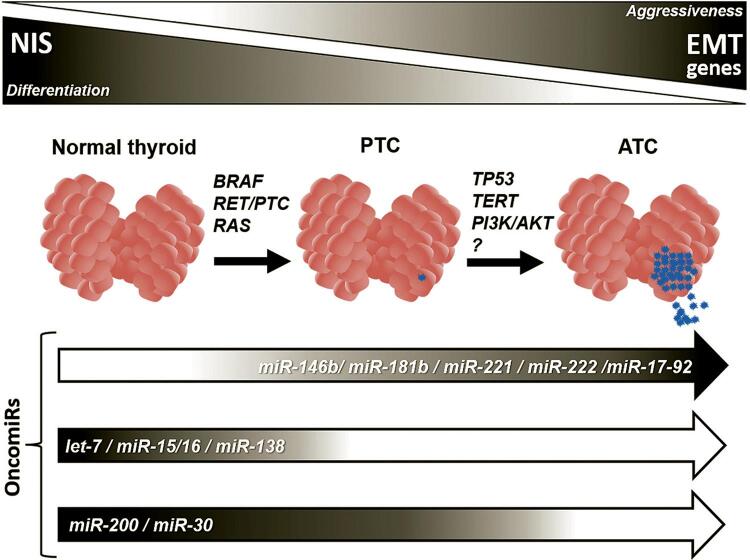
Oncogene activation induces thyroid cancer and leads to deregulation of miRNA expression (loss or gain of expression). The loss of NIS expression is key for the thyroid dedifferentiation and impairment of responsiveness to radioiodine therapy. The activation of epithelial-to-mesenchymal transition (EMT) genes expression contributes to cancer progression and aggressive phenotype. Modified from Fuziwara & Kimura (14).

Normally, the thyroid gland expresses a specific constitutive pool of miRNAs that includes *let-7* isoforms (*let-7a*, *let-7b*, *let-7c*, *let-7d*, *let-7e*, *let-7g*, and *let-7i*), *miR-15/miR-16 cluster*, *miR-30a/miR-30c/miR-30d*, *miR-125b*, *miR-200c*, *miR-99b*, and *miR-138* (16). Strikingly, several of these thyroid-specific miRNAs are deregulated in thyroid cancer, which indicates that oncogenic activation could interfere with thyroid cells’ function by changing the levels of the miRNAs (Figure 1). However, some miRNAs that are not expressed in normal thyroid cells are activated in thyroid cancer. One of the hallmarks of miRNAs in PTC is *miR-146b*, consistently increased in PTC, and which expression correlates with a poor prognosis (5,17).

The results of a *Dicer1* knockdown study highlight the importance of miRNAs for thyroid cells’ differentiation (18). Primary miRNA is transcribed and, sequential cleavage processes that occur in the nucleus and cytoplasm generate mature miRNA. The main protein involved in the final step of miRNA biogenesis is the ribonuclease DICER (19). Indeed, *Dicer1* knockdown resulted in severe downregulation of thyroid cell-differentiation genes *Nis*, *Tpo*, *Tg*, *Nkx2-1* and *Pax8* in normal rat thyroid follicular cell line PCCl3 *in vitro* (18).^.^ Additionally, across three studies of *Dicer1* transgenic knockout mice, a similar effect occurred in the thyroid gland, with decreased expression of thyroid-differentiation markers, severe impairment of thyroid-hormone secretion and hypothyroidism, and – interestingly – loss of thyroid histological organization (20,21). This global downregulation of miRNAs caused by the *Dicer1* knockout indicates that the miRNAs’ regulatory network also controls the thyroid gland’s functioning. Indeed, the results of an in silico analysis (using a bioinformatics algorithm for target prediction) reveal that several miRNAs could target thyroid-differentiation genes and TFs (15).

Interestingly, thyroid TF PAX8 can drive the expression of *miR-146b* by binding to *miR-146b* promoter; similarly, the TF NKX2-1 does so by binding to *miR-532-5p* promoter (22,23); this indicates the existence of an intricate regulatory network between thyroid TFs and miRNAs for the control of thyroid follicular cells’ function. Moreover, to date, a few miRNAs are directly associated with the regulation of thyroid differentiation genes. Among them, the 3’ strand of *miR-146b* (*miR-146b-3p*) targets *NIS* mRNA in *NIS*-expressing cells and thus reduces radioiodine uptake (23). Similarly, an in silico analysis led to the discovery of *miR-339-5p* as a novel regulator of *NIS* expression, impacting thyroid follicular cells’ function (24).

In addition, miRNA could also regulate thyroid differentiation in an indirect manner. For example, *miR-106a* is upregulated in the serum of patients who have thyroid-cancer metastases in the lung that are not avid for radioiodine (25). Similarly, *miR-106a* regulates the expression of retinoic acid receptor beta, (*RAR*β) which influences thyroid cells’ differentiation. The overexpression of *miR-106a* in the PTC cell line increases cell viability and invasion but reduces apoptosis; the opposite occurs when inhibiting *miR-106a* in ATC cell lines. Moreover, targeting *miR-106a* with antagomir increases the expression of *NIS* and *TSHR* while sensitizing ATC cells to radioiodine treatment (25). Interestingly, *miR-146b* regulates the expression of RARβ (26) and serum *miR-146b* levels are also associated with thyroid tumors poor prognosis (27).

## TGFβ SIGNALING AND THYROID FUNCTION

Transforming Growth Factor β (TGFβ) signaling is an important pathway in thyroid cells’ function and homeostasis. Classically, TGFβ signaling is a tumor suppressor in normal epithelial cells (e.g., thyrocytes), as it exerts an antimitogenic effect; nevertheless, the cumulative evidence shows that it plays additional roles in thyroid-cell differentiation and in the epithelial-to-mesenchymal transition (EMT) process, and promotes malignant progression of cancer.

The cascade of TGFβ signaling activation initiates when the TGFβ isoforms (TGFβ1, TGFβ2, and TGFβ3) are bound to the membrane-receptor serine kinases of the TGFβ family. Ligand binding forms a heterodimeric complex that comprises two type-II (TGFBR2) and two type-I (TGFBRI) receptors. Then, TGFBR2 phosphorylates TGFBRI for at multiple serine threonine residues in the N-terminal region. Activated TGFBRI recruits and phosphorylates SMAD2 and SMAD3 proteins (R-SMADs), leading to the formation of the SMAD2/3 and SMAD4 heterotrimeric complex. Subsequently, this complex translocates to the nucleus and binds to the SMAD-binding element of the DNA in order to modulate the transcription of target genes such as cell-cycle inhibitors (e.g., p21^CIP1^ and p15^INK4^). SMAD7, a negative regulator of this pathway, interacts with activated receptors and R-SMADs, thus suppressing translocation. Moreover, SMURF1/2 exerts posttranslational regulation by ubiquitination of SMAD proteins and receptors, leading to ubiquitin–proteasome degradation (28).

Thyroid tumors express TGFβ/SMAD signaling components (29,30), unlike other types of cancer (which typically harbor genetic alterations in this pathway). Instead, signaling transduction deregulation is related to the modulation of its components through oncogene-induced posttranscriptional changes, as well as through posttranslational modifications such as ubiquitination, or through the signaling transduction inhibition by SMAD7. Indeed, low levels of SMAD4 can occur in PTC cell lines, thus causing refractoriness to TGFβ’s cytostatic effect (29). Acute BRAF^V600E^ activation in normal thyroid follicular cells leads to both downregulation of SMAD4 and refractoriness to TGFβ1-induced cell-cycle arrest via the activation of the *miR-17-92* cluster and *miR-146b*. Moreover, the blockage of these miRNAs using antisense oligonucleotides with locked nucleic-acid (LNA) modifications (antagomirs) results in the recovery of SMAD4 protein levels and increased responsiveness to TGFβ1’s antimitogenic effect (31,32).

In addition to the classical antiproliferative effect of the TGFβ/SMAD pathway (activation of cell-cycle arrest), TGFβ is a well-known inhibitor of thyroid differentiation and growth (33,34). The treatment of FRTL5 cells (a normal rat thyroid follicular cell line) with exogenous TGFβ1 leads to downregulation of *Nis*, *Tpo* and *Tg*. Moreover, the disruption of TGFβ signaling transduction (in this case, using a SMAD4 dominant negative protein in FRTL5 cells) also results in the loss of differentiation — to a similar extent as for TGFβ-treated cells (35) The molecular basis for this regulation is attributed to the binding of SMAD3 to PAX8, which in turn impairs PAX8’s action on the *Nis* upstream enhancer and leads to *Nis* transcriptional repression (36). Indeed, TGFβ impairs *Tg* transcription by reducing the levels of PAX8, thus diminishing its DNA-binding activity (37). Interestingly, in normal thyroid cells *in vitro*, BRAF^V600E^ activation also represses *NIS* expression via the increased secretion of TGFβ (38). Additionally, TGFβ signaling can regulate the expression of *NKX2-1* via a miRNA loop, wherein TGFβ treatment induces *miR-365* which targets *NKX2-1*, while *miR-365* overexpression induces TGFβ secretion (39).

The high expression of TGFβ1 occurs in the invasive fronts of PTC, correlating with the loss of thyroid differentiation (38); and it is also observed in poorly circumscribed PTC that shows high levels of TGFβ in the tumor-periphery invasive front; on the other hand, in well-circumscribed PTC, the center of the (noninvasive) tumor shows inhibition of TGFβ signaling (high SMAD7 levels) (40).

## TGFβ AND MIRNAS IN EMT REGULATION

TGFβ signaling plays a dual role, and its function depends on the cellular context. Although, in normal epithelial cells, its signaling induces the expression of the p21 and p27 cell-cycle inhibitors to block cell proliferation, in cancer cells, TGFβ promotes EMT, which is a hallmark for cancer progression (41). In a recent study, Xu and cols. (42) shed some light into the molecular mechanism that breast-cancer cells use to reprogram the TGFβ antiproliferative signaling and to promote EMT and metastasis; the results indicate that YWHAZ (14-3-3ζ) activation is key to overcoming the cytostatic effect of TGFβ because it blocks the p53/SMAD association and induces GLI2/SMAD interaction in breast cancer bone metastasis. YWHAZ represses the expression of SFN (14-3-3σ), which is necessary to stabilize p53 protein levels, and consequently leads to a loss of TGFβ-induced p21 expression (42).

EMT is a general term that is used to describe the mechanism through which a polarized cell undergoes a series of molecular changes to acquire a mesenchymal phenotype, which enhances the migratory and invasive potential and improves the extracellular matrix’s secretory ability (43). EMT can be further classified into 3 types: type I, which occurs during embryo implantation and gastrulation; type 2, which is activated by injury or inflammation in normal tissues; and type 3, which is activated during cancer progression (43).

EMT is classically activated by TGFβ-signaling transduction via SMAD proteins; this leads to transcriptional changes that downregulate epithelial genes (E-cadherin, cell-to-cell junctions, and cell-polarity genes), that induce mesenchymal genes (N-cadherin and extracellular-matrix interaction genes), and that lead to cytoskeleton remodeling (44). This process depends on the activation of three families of EMT TFs: SNAIL, ZEB, and bHLH (which includes Twist). ZEB1, ZEB2, SNAI1, SNAI2 (SLUG), and TWIST1 are the master EMT TFs as they autoregulate each other and cooperatively repress the transcription of E-cadherin (*CDH1* gene), which is the hallmark of EMT – all while orchestrating changes to the expression profile that lead to the EMT phenotype shift (41).

In a zebrafish model model, BRAF^V600E^-induced PTC led to EMT activation and thyroid-gland disorganization via the TF TWIST2 (45). Moreover, BRAF^V600E^ activation in the thyroid gland of transgenic mice (Tg-Braf) generated PTC with regions of poorly differentiated thyroid cancer at the late stage (5 months) (46). A microarray gene-expression profile comparison of PTC and poorly differentiated tumors in transgenic Tg-BRAF mice revealed a reduction of cell-adhesion genes such as E-cadherin and an increase in the intermediate filament vimentin. Indeed, the TGFβ-signaling pathway was activated in these poorly differentiated tumors, which show high levels of pSMAD2, repression of E-cadherin, and upregulation of the vimentin protein (46). Interestingly, TGFβ-signaling activation is dependent on MAPK signaling, as a treatment with U0126 (a MEK inhibitor) blunts SMAD2 phosphorylation and blocks TGFβ-induced genes in BRAF-induced, PTC-derived primary cells (46).

Furthermore, the signaling activation of MAPK using BRAF^V600E^ induces the ETV5 TF, which subsequently modulates TWIST1 expression transcriptionally to promote EMT in thyroid-cancer cells (47). Conversely, in a human PTC cell line, *ETV5* knockdown attenuated this phenotype and reduced cell migration, invasion, and proliferation. Moreover, high nuclear levels of TWIST1 in PTC cell lines are positively correlated with metastasis and are associated with the NFκB-signaling pathway activation as the knockdown of NFkB blocks TWIST1 upregulation (48).

There is an intricate network of regulation between miRNA and TGFβ signaling. On the one hand, miRNAs can target the TGFβ pathway’s components, thus acting as oncomiRs (miRNAs that are deregulated during oncogenesis and cancer progression), as shown in Table 1. On the other hand, TGFβ signaling can modulate miRNA expression through two mechanisms: (a) influencing the transcriptional activity of miRNA genes by binding the pSMAD complex to the promoter region of these genes at the sites of the SMAD-binding element or (b) modulating the processing of precursor miRNA (pre-miRNA) into mature miRNA. For example, the binding of SMAD2/3 to a SMAD-binding element’s region in the primary miRNA stabilizes the microprocessor complex (DROSHA, DGCR8, and p68) and induces the maturation of pre-mir-21 and pre-mir-199a in the vascular smooth muscle (49).

**Table 1:** Bioinformatic prediction of microRNAs that target mRNA related with TGFβ signaling pathway

mRNA	microRNAs^*^						
***TGFBR1***	*miR-181-5p*	*miR-133a-3p*	*miR-142-3p*	*Let-7*	*miR-183-5p*	*miR-101-3p*	*mir-208-3p*
***TGFBR2***	*miR-17/ 20-5p*	*miR-19-3p*	*miR-21-5p*	*miR-302-3p*	*miR-130-3p*	*miR-142-5p*	*miR-144-3p*
***SMAD2***	*miR-18a-5p*	*Let-7-5p*	*miR-200b/c/429*	*miR-142-5p*	*miR-128-3p*	*miR-155-5p*	*miR-181-5p*
***SMAD3***	*miR-145-5p*	*miR-15/16*	*miR-129-3p*	*miR-23-3p*	*miR-216-5p*	*miR-143-3p*	*miR-18-5p*
***SMAD4***	*miR-146b-5p*	*miR-17*	*miR-19*	*miR-18a*	*miR-142-3p*	*miR-34*	*miR-205-5p*
***SMAD7***	*miR-21*	*miR-181-5p*	*miR-182-5p*	*miR-17/20*	*miR-15/16*	*miR-200b/c*	*miR-25*
***SMURF1***	*miR-19-3p*	*miR-15/16*	*miR-137*	*miR-25-3p*	*miR-142-5p*	*miR-200bc*	*miR-153-3p*
***SMURF2***	*miR-19-3p*	*miR-15/16*	*miR-137*	*miR-124-3p*	*miR-142-3p*	*miR-200bc*	*miR-130-3p*
***ARKADIA (RNF111)***	*miR-19-3p*	*miR-15/16*	*miR-21-5p*	*miR-142-3p*	*miR-144-3p*	*miR-155-5p*	*miR-9-5p*

* Prediction based on TargetScanHuman Release 7.2 (http://www.targetscan.org/vert_72/).

**Figure 2:**
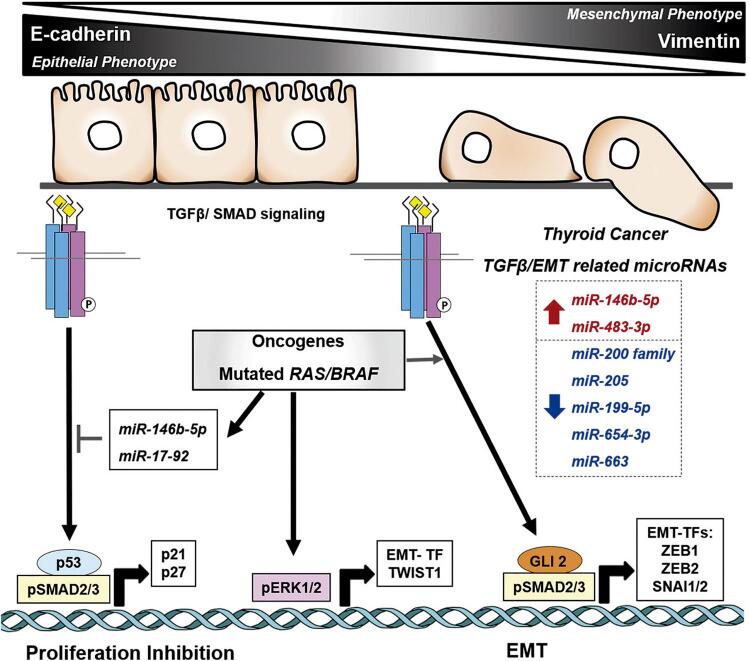
TGFβ signaling exerts a dual role in thyroid follicular cells. In normal cells, TGFβ induces antimitogenic effect by induction of p21/p27, while upon activation of MAPK oncogenes and cell transformation, TGFβ promotes EMT and invasion via induction of EMT transcription factors (EMT-TFs). At the right side, the list of deregulated miRNAs that contribute to TGFβ-induced EMT in aggressive thyroid cancer, leading to loss of epithelial phenotype (E-cadherin) and acquisition of mesenchymal phenotype (Vimentin).

The discovery of miRNA networks has shed light on EMT, as these networks can regulate several features of the EMT signaling transduction, from the proteins that act as activators (such as TGFβ and its pathway) to the EMT TFs (Figure 2). The results of a seminal study show that activation of the protein tyrosine phosphatase Pez in combination with TGFβ signaling significantly silences both the *miR-200* superfamily and *miR-205* (50) At the same time, when different human cancer cell lines are stratified according to the levels of epithelial (high in E-cadherin) and mesenchymal (high in vimentin) gene signatures, the miRNA signature of the mesenchymal-phenotype cancers show strong downregulation of the *miR-200* family (51). In humans, the *miR-200* family is composed of 5 miRNAs that share 2 seed regions (*miR-200a/141* and *miR-200b/c/429*) across independent clusters: chr12 (*miR-200c/141*) and chr1 (*miR-200a/b/429*) (50).

The functional investigation of *miR-200s* shows that, on the one hand, *miR-200* expression blocks EMT by targeting the ZEB1/ZEB2 TFs that downregulate E-cadherin, but that, on the other hand, ZEB1 transcriptionally inhibits *miR-200* expression in a negative feedback loop (50,51). Indeed, the *miR-200* family is downregulated in anaplastic thyroid carcinoma (52). The results of a microarray gene-expression analysis reveal that the *miR-200c/141* and *miR-200b/a/429* clusters are severely repressed in ATC cells, as compared to normal thyroid cells and cells from other types of thyroid cancer such as PTC and follicular thyroid cancer (52). Interestingly, *miR-200c* expression (together with the *let-7* family) is among the 10 most abundant miRNAs in normal thyroid glands (16), which indicates that the *miR-200* family plays an important role in thyroid biology. In normal thyroid cells, the activation of the Epidermal Growth Factor (EGF) signaling pathway inhibits the *miR-200* family and induces EMT, with a loss of E-cadherin. On the other hand, in the anaplastic thyroid carcinoma cell line and in xenotransplant tumors, blockage of EGFR signaling restores *miR-200* expression and also induces both the mesenchymal-to-epithelial transition and E-cadherin upregulation (53). Similarly, *miR-205* downregulation is associated with TGFβ-induced EMT and cooperates with *miR-200* family (50). In anaplastic thyroid cancer, *miR-205* overexpression blocks EMT and cell invasion, and restores E-cadherin expression. Moreover, *miR-205* inhibits VEGF-A secretion and angiogenesis *in vitro* and tumor growth mouse xenotransplant (54).

Recent data further have indicated that a new set of miRNAs regulate the thyroid-cancer EMT process (Figure 2). For instance, in PTC samples, *miR-199-5p* is downregulated and inversely correlated with *SNAI1* mRNA levels. In thyroid-cancer cell lines, the restoration of *miR-199-5p* levels leads to SNAI1 repression, which in turn leads to the inhibition of N-cadherin and vimentin, as well as the induction of E-cadherin (55). Indeed, *miR-199-5p* represses SNAI1 by binding to the mRNA’s 3’ untranslated region, and *SNAI1* knockdown reduces cell invasion and tumor growth in xenotransplants. This indicates the important role of *miR-199-5p* as an EMT inhibitor. Moreover, in PTC, *miR-663* is a target of TGFβ1-induced EMT and is downregulated. Ectopic expression of *miR-663* in the BCPAP and IHH4 PTC cell lines causes the restoration of E-cadherin levels in a similar way as in the silencing of TGFβ1 (56). In addition, *miR-654-3p* is downregulated in human PTC samples and in thyroid-cancer cell lines. BRAF^V600E^oncogene-induced mouse PTC suppresses *miR-654-3p* levels in the late stage, inversely correlating with increases in the EMT TFs *Zeb1, Zeb2, Snai1* e *Snai2* (57). The reintroduction of *miR-654-3p* in thyroid-cancer cells reduces cell migration and decreases *Snai2* transcription, while also increasing E-cadherin levels.

Additionally, miRNA upregulation can promote thyroid cancer EMT. The results of a recent study show that *miR-483-3p* plays a role in regulating the *PARD3* polarity gene in ATC, thus enhancing the EMT process (58). Indeed, *miR-483-3p* is highly expressed in ATC cell lines, and it can be induced via TGFβ treatment to target *PARD3* mRNA. The loss of PARD3 occurs in thyroid cancer (as compared to a nontumoral counterpart); and low levels of *PARD3* are inversely correlated with *miR-483-3p* expression. Blocking *miR-483-3p* using antagomirs inhibits TGFβ-induced cell invasion and prevents PARD3 downregulation. Interestingly, PARD3 rescue in ATC cells leads to the blockage of TGFβ-induced effects via the maintenance of E-cadherin levels and the inhibition of vimentin (58).

Hardin and cols. reported an additional loop of TGFβ-miRNA interaction, showing upregulation of *miR146-5b* expression in PTC cell line exposed to TGFβ1 (59). Moreover, the treatment of PTC cells with TGFβ1, both *in vitro* and in a xenograft model, downregulates E-cadherin and induces SLUG, SNAI, TWIST, and vimentin (59). Indeed, *miR146-5p* is one of the most highly expressed miRNAs in BRAF-mutated PTC (5,17) and it targets zinc and ring finger 3 (ZNRF3), which is a modulator of Wnt/β-catenin signaling. In PTC, the high expression of *miR146-5p* suppresses ZNRF3 and enhances the expression of EMT markers (60).

Interestingly, distant metastases that arise from carcinomas usually show a well-differentiated epithelial phenotype; this hints at another interesting aspect of EMT: its reversibility in the mesenchymal-to-epithelial transition. In a seminal study, Ocana and cols. (61) found that TGFβ-induced EMT involves the activation of the TF paired-related homeobox-1 (PRRX1), which is expressed in the normal development of somites’ mesenchymal cells. PRRX1 overexpression is sufficient to induce EMT in MDCK cells; this leads to the mesenchymal phenotype, including invasion, loss of E-cadherin, expression of vimentin, and activation of some EMT TFs (61). However, according to the results of a tail-vein injection assay, the cancer cells that overexpress PRRX1, although relatively invasive, cannot colonize distant sites; this shows that, the loss of PRRX1 is essential for lung colonization, as it reverses the EMT via the recovery of the epithelial phenotype (61).

## PERSPECTIVES

Recent therapeutic strategies have been intended to improve radioiodine uptake in advanced thyroid cancer through the use of MAPK signaling inhibitors. However, resistance to these inhibitors is somewhat common, so there is an urgent need to develop adjuvant therapies. For example, a combinatorial therapy with MAPK-signaling inhibitors and histone-deacetylase inhibitors showed a synergistic effect in reexpressing the *NIS* gene and in recovering the radioiodine trapping in BRAF^V600E^ thyroid-cancer cell lines (62). On the other hand, researchers have investigated the efficacy of the TGFβ pathway inhibition in preclinical and clinical trials using several TGFβ antagonists, including TGFβ antibodies, antisense oligonucleotides, and receptor-kinase inhibitors. In cancer, this inhibition could attenuate the TGFβ-dependent EMT; however, due to the pleiotropic functions of TGFβ signaling – which regulates many normal physiological functions and various steps of cancer progression – the effects of TGFβ inhibitors in cancer therapy remain unpredictable (63). Furthermore, the progressive cascade of events in cancer metastasis is activated by TGFβ, which recruits several cell types in the tumor microenvironment – not just primary tumor cells but also stromal and immune cells (64). Thus, developing a strategy to target all cell types at once is a challenge in the development of any targeted molecular therapy.

The picture that emerges from this evidence is that modulating deregulated miRNAs, in combination with the conventional or new generation of inhibitors, could help to reestablish thyroid cells’ iodine-trapping function. This treatment could thus act as an adjuvant therapy in iodine-refractory thyroid cancer and could influence the EMT phenotype. For example, *miR-146b* is upregulated in a differentiated thyroid-carcinoma-cell model in which radioiodine resistance is acquired after radioiodine exposure, leading to the downregulation of the thyroid-differentiation genes (65). Interestingly, the inhibition of *miR-146b* can restore thyroid differentiation and radioiodine trapping via the upregulation of NIS levels; this inhibition thus exerts a global effect in cell biology by reducing cell viability and inducing apoptosis. Conversely, reexpression of the *miR-200* family, as well as of *miR-205* and other downregulated miRNAs, could improve thyroid-cancer prognosis by blunting the EMT and metastasis processes. In this regard, the strategy of targeting miRNAs could have a potential broad effect as those miRNAs control a plethora of genes.
